# Burn Pain Management at Burn Unit of Yekatit 12 Hospitals, Addis Ababa

**DOI:** 10.1155/2018/1092650

**Published:** 2018-07-04

**Authors:** Negashu Dadi Mengistu, Mohammed Suleiman Obsa, Leulayehu Akalu Gemeda

**Affiliations:** ^1^Department of Anesthesia, Wolaita Sodo University, Wolaita Soddo, Ethiopia; ^2^Department of Anesthesia, Jimma University, Jimma, Ethiopia; ^3^Department of Anesthesia, Addis Ababa university, Addis Ababa, Ethiopia

## Abstract

**Background:**

Burn pain is a unique and complex challenge for all health professionals and the patients. The only way to insure quality burn pain relief is the patient's self-report. Thus, assessment of severity of pain and its associated factors are important in treatment plan.

**Objective:**

To assess severity of burn pain and associated factors in Yekatit 12 Hospitals burn unit from January 1, 2017, to March 30, 2017.

**Methods:**

Hospital based cross sectional study design was conducted. Data was collected by using structural questionnaires from all eligible patients, those admitted to burn unit of Yekatit 12 Hospital during the study period. Severity of burn pain was assessed by using numerical, facial, and behavioral pain scale measurement tools. Pain assessment scale was deployed for patients by data collectors before medication and dressing change. Multivariate logistic regression analysis was conducted to identify significant predictors based on p value less than 0.05 with 95% confidence interval.

**Results:**

A total of 62 burn patients were included in the study. 87.1% of the patients feel severe pain and 12.9% of the patients feel moderate pain. Pediatrics age groups were about 11 times more likely to feel severe pain than adults, and patients with TBSA ≥ 25% were about 8 times more likely to feel severe burn pain than those with TBSA < 25% [AOR = 7.773; CI 1.184, 51.043] (P = 0.033).

**Conclusion and Recommendation:**

Majorities of burn patients had severe pain and burn pain was not appropriately treated. Therefore, appropriate pain management was strongly recommended.

## 1. Introduction

Burns are extremely painful and the most common causes of distress to the patients [1]. Burn injuries are classified as one of the most devastating of all injuries and major global public health crisis. Approximately 90% of burns occur in low to middle income countries. It is a serious pathology, potentially leading to severe morbidity and significant mortality and it has also a considerable health-economic impact [2].

There are different factors that affect the severity of burn pain. Burn patient with large area full-thickness burn suffers more pain than patient with small area full-thickness burns. The experience of pain is largely related to patient development age, prior experience with pain cultural characteristics of the patient, degree of burn, source of burn, duration of burn, and management gaps [3].

Unrelieved burn pain has been described as a significant public health problem. The long-term risk of undertreated pain is associated with the development of chronic pain, depression, and Posttraumatic Stress Disorder (PTSD) [4]. A successful treatment requires careful assessment of its nature, understanding the different types and patterns of pain, and knowing the best treatment. A good initial assessment serves as a baseline to evaluate the results of subsequent interventions [5].

Pain scales are used to monitor pain by using point scale of 0–10. People have a means to communicate their pain intensity and clinicians have means to track it, just as they would keep track of other vital signs, like blood pressure, respiration, and pulse rate. Even though pain is a subjective experience that cannot be verified by traditional diagnostic methods, it cannot be effectively treated unless it is measured [6].

According to National Institute for Health, the most commonly used pain assessment methods are numerical and behavioral assessment scale. Numerical scales can be used for both adults and children who can express themselves where 0 is having no pain, 1-3 mild pain, 4-6 moderate pain, and 7-10 severe pain. For children and adult patients who are unable to provide a self-report of pain, behavioral assessment scale can be used to assess pain [7].

WHO “pain ladder” guidelines recommend that pain is treated according to its severity. Mild pain can be treated by nonopioid drugs like paracetamol and anti-inflammatory drugs. Then, if complete pain relief is not achieved, a weak opioid such as tramadol is added to the existing nonopioid regime. If this also becomes insufficient, a weak opioid is replaced by a stronger opioid, such as morphine, fentanyl, and pethidine while continuing the nonopioid therapy. If the initial presentation is severe pain, this stepping process should be skipped and a strong opioid should be started immediately in combination with a nonopioid analgesic [8].

Nonpharmacological therapy is an important measure complementary to medication to manage pain and anxiety in burn patients. It should be initiated as early as possible in order to prevent the development of anxiety, which can prolong the cycle of pain [9].

## 2. Method and Materials

### 2.1. Study Area and Study Period

The study was conducted in burn unit of Yekatit 12 Hospital, Addis Ababa, Ethiopia, from January 1, 2017, to March 30, 2017. Yekatit 12 Hospital is one of the hospitals under Addis Ababa City Administration Health Bureau that has been giving routine health services for Addis Ababa and other referral cases from different regional states of Ethiopia. The hospital provides services for a population of approximately 4 million people. It has 9 departments and 6 units and has 265 beds. It has been the main referral hospital for treatment of burns patients for many years. The burn unit has 19 beds, 12 of them for adults and 7 for pediatrics. 

*Study Design.* Hospital based cross sectional study design was employed. 

*Source Population.* All burn patients in Yekatit 12 Hospital burn unit constituted the source population. 

*Study Population. *Selected burn patients who fulfill inclusion criteria and admitted to burn unit of Yekatit 12 Hospital during the study period were the study population. 

*Sample Size Determination and Sampling Technique.* Census was used to include all burn patients in Yekatit 12 Hospital burn unit. 

*Inclusion Criteria.* Inclusion criteria were all eligible burn patients who provide consent. 

*Exclusion Criteria*. Very critically ill burn patient, patients who refused to give consent, and patients who were referred for further management were excluded from the study. 

*Data Collection Tools and Procedure. *Data was collected using pretested structured questionnaires by two degree holder nurses and supervised by one M.S. anesthetist. A numerical, facial, and behavioral pain scale measurement tool was employed to assess the severity of burn pain. Numerical assessment scale is used for adults and older children (>12 years), facial pain assessment scale is used for children between 4 and 12 years and behavioral pain assessment scale is used for children 1–4 years old. Pain assessment scale was deployed for patients by data collectors before medication and dressing change. The data was collected by chart review and patient's interview. Each of the five categories (FLACC scale), F = face, L= legs, A = activity, C = cry, and C = consolability, is scored from 0 to 2. This results in a total score of 0–10. Muscle tone was assessed in patients with spinal cord lesion and hemiplegia of unaffected side (zero = no evidence of pain; 1-3 = mild pain; 4-6 = moderate pain; 7-10 = severe pain (Erdek and Pronovost, 2004)).

**Figure 1:**
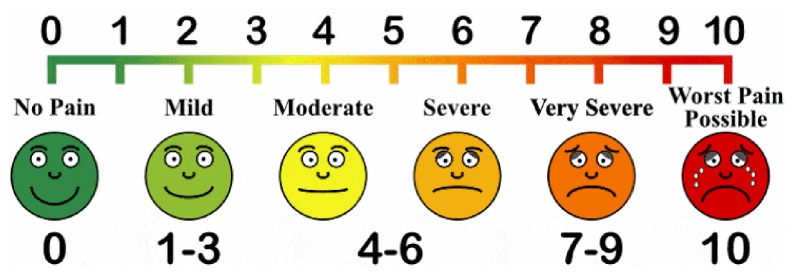


**Figure 2:**
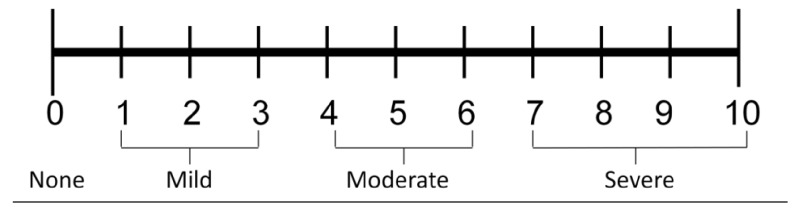


1. Faces Pain Scale-Revised for children 4–12 years (Figure 1).
2. The Numeric Pain Rating Scale for adults and older children (>12 years) (Figure 2) (Mc Caffery, M., Beebe, A. et al. (1989). Pain: clinical manual for nursing practice, Mosby, St. Louis, MO).

*Data Quality Assurance.* Data was collected by using structured questionnaire, which addresses the objective of the study. The questionnaire was prepared in English first and translated into the local language, Amharic and Afan Oromo, and again back into English to ensure the consistency of the question. Pretest was done on 5% of the sample size. Data collectors and supervisors were trained on each of the items included in the study tools. During data collection, regular supervision and follow-up were made. 

*Data Analyzing and Processing.* The data was entered on Epi Info version 7 and exported to SPSS version 20 computer program for analysis. Descriptive statistics were used to summarize data, tables, and figures for display results. Bivariate and multivariate analysis were used to see the effect of independent variable on outcome variable. Variables which are significant on bivariate analysis at p value less than 0.2 were taken to multivariate analysis. In multivariate analysis P value of less than 0.05 was used as a cut-point for presence of association. Strength of association was measured by 95% confidence interval and odd ratio. 

*Ethical Considerations.* Ethical clearance and approval were obtained from ethical review committee, Anesthesia Department, Addis Ababa University. Permission was obtained from Yekatit 12 Hospital. Informed verbal consent was secured from all study participants. The obtained data was only used for study purpose. Confidentiality and anonymity were ensured.

### 2.2. Operational Definition

*Acute Pain.* It is usually a direct result of tissue trauma and the severity of which reflects the degree of tissue damage

*Burn Pain.* It is the actual tissue damage that resulted from burn injury. 

*Chronic Pain.* Malignant or nonmalignant pain that exists beyond its expected time frame for healing or where healing may not have occurred. It is persistent pain that is not amenable to routine pain control methods.

## 3. Results

A total of 62 patients were included in the study over three months of the study period with 38 females and 24 males (female  :  male ratio of 1.6:1). Among them, the highest number of 41 (66.1%) is found in the age group of 1–14 years and 21 (33.9%) are in the age groups of ≥15 years. The mean age of respondents was 12.84 with the SD of 11.05 and minimum 1 year and maximum 40 years (Table 1).

### 3.1. Severity of Burn Pain and Medication Given

Out of 62 of the patients, 41.9% were Amhara, 35.5% were Oromo, 11.3% were Gurage, 6.5% were Tigre, and 4.8% were others (Afar and Wolaita) (Table 2). Majority of the patients (87.1%) were feeling severe pain, and 12.9 were feeling moderate pain. Among patients who felt severe pain the majority of them (48.4%) took paracetamol, 30.6% took tramadol, and the remaining 8.1% took nonsteroidal anti-inflammatory drugs (diclofenac).

### 3.2. Distribution of TBSA, Degree, Source, and Duration of Burn

The major causes of burn were hot water 35.5%, electricity 30.6%, fire wood 16.1%, gas 11.3%, and others (stove) 6.5%. Majority of the burns (56.5%) were second-degree burns and the rest (43.5%) were third-degree burns. Majority total surface area of burn was >25% which accounts for 38.7%. Majority of burns duration (62.9%) were within one month and the rest (17.7%) were between one and two months, 12.9% were more than three months, and 6.5% were between one and two months (Table 3).

### 3.3. Factors Associated with Severity of Burn Pain

In multivariate analysis, where 95% CI for the adjusted odds ratios were calculated among these variables, severity of pain is statistically significant with ages (P = 0.039) and those patients whose TBSA ≥ 25% (P = 0.033) of the study groups. Those study participants whose age ≥ 15 years are 0.09 times less likely to feel severe burn pain than 1–14-year-old patients (AOR = 0.090; CI 0.009, 0.883). Those study subjects whose TBSA ≥ 25% are 7.773 times more likely to feel severe burn pain than those TBSA < 25% of patients (AOR = 7.773; CI 1.184, 51.043) (Table 4).

## 4. Discussion

In this study also females feel more severe burn pain than male patients, but the difference was not statistically significant (P = 0.102) which is comparable with the above study. The study done in South Korea shows that female patients reported more severe pain than male patients, but the difference was not statistically significant (*χ*2 = 3.106, P = 0.082) [10].

This study also shows that the study participants whose age ≥ 15 year are 0.09 times less likely to feel severe pain as compared to those 1–14-year-old patients (AOR = 0.090; CI 0.009, 0.883); i.e., pediatrics age groups are 11.1 more likely to feel more severe pain than adults. According to Blankenburg M et al., there is a difference between adults and children in pain sensitivity. Children are more sensitive to pain stimuli compared to adults. Sex differences were minimal, during childhood [11].

According to Miller C NS, severity of burn pain depends on degree of burn and percentage of total burns surface area (TBSA). Usually 3^rd^-degree burns are the least painful as compared to 2^nd^-degree burns, and this is because the nerve endings have been burned and patients with 2nd-degree burns experience greater pain as the ultrasensitive nerve endings are exposed and as TBSA increased the patients suffer more intense pain as compared with patients with small body surface area burned [12].

This study also revealed that burn patients with high percentage of total surface area (TBSA ≥ 25%) are 7.773 times more likely to feel severe pain than those with TBSA < 25% (AOR = 7.773; CI 1.184, 51.043). Second-degree burn patients also feel more severe pain than third-degree burn patients but the difference was not statistically significant (P = 0.072).

The study done in India General Hospital, in 2015, showed that the percentage of patients who were suffering from pain increased with the level of education from 8% in the illiterates to 19% in the partially educated and then 46% in the university graduates. This is due to increased awareness levels, and they give more attention to pain which intensifies it [13].

This study also shows that among adult patients severity of burn pain is increased with the level of education from 9.8% in the illiterates to 14.3% in the partially educated and then 19.1% in the university graduates which is comparable. According to WHO “pain ladder” guideline of pain management, mild pain should be treated with nonopioid drug like paracetamol and NSAIDS. Moderate pain is treated with weak opioid such as tramadol. Severe pain is treated with strong opioids such as morphine, fentanyl, and pethidine while continuing the nonopioid therapy [8].

In this study even though majority of the patients (87.1%) were feeling severe pain, most of them (48.4%) took paracetamol and 30.6% took tramadol, which exacerbate the intensity of burn pain. This may be due to management gaps (skill or resource).

The study, conducted in Toronto, showed that, in addition to gender, ethnicity was also an important factor for pain. The differences were found in white Americans and African Americans living in the same social and geographical environment. African Americans felt more pain than white Americans [14]. However, in contrast to the above study, in our study there is no association of pain with ethnicity. This may be because our study was conducted in the same race.

## 5. Conclusion and Recommendation

Majorities of burn patients had severe pain and there was inadequate management of burn pain. Therefore, health care providers should treat burn patients according to pain management protocol as mentioned in WHO pain ladder of pain management, hospital administration should provide adequate resources needed for burn pain management as there is lack of adequate resources to manage burn pain, and patients were recommended to report severity of burn appropriately so that appropriate drug can be used for the treatment of burn pain.

## Figures and Tables

**Table 1:** Age and sex distribution of burn patients in Yekatit 12 Hospital, Addis Ababa, Ethiopia, from January 1, 2017, to March G.C. (N = 62).

Variables	Categories	Frequency	Percentage	Mean ± SD
Age	1-14 years	41	66.1	12.84 ± 11.05
	≥15 years	21	33.9	
	Total	62	100.0	

Sex	Male	24	38.7	
	Female	38	61.3	
	Total	62	100.0	

**Table 2:** Distribution of severity of burn pain and medication given to the patients, in Yekatit 12 Hospital, Addis Ababa, Ethiopia, from January 1, 2017, to March 30, 2017 G.C. (N = 62).

Severity of pain	Medication						Total	
	NSAIDS		Paracetamol		Tramadol			
	Frequency	Percent	frequency	percent	frequency	percent		
Moderate	1	1.6%	2	3.2%	5	8.1%	8	12.9%

Severe	5	8.1%	30	48.4%	19	30.6%	54	87.1%

Total	6	9.7%	32	51.6	24	38.7	62	100.0%

**Table 3:** Distribution of TBSA, degree, source, and duration of burn among patients at Yekatit 12 Hospital, Addis Ababa, from January 1, 2017, to March 30, 2017, G.C. (N = 62).

Variables	Categories	Frequency	percentage
TBSA	≤25%	15	24.2
	>25%	47	75.8
	Total	62	100.0

Degree of burn	Second degree	46	74.2
	Third degree	16	25.8
	Total	62	100.0

Source of burn	hot water	22	35.5
	Electricity	19	30.6
	fire wood	10	16.1
	Gas	7	11.3
	Others	4	6.5
	Total	62	100.0

Duration of burn	0-30 day	39	62.9
	31-60 day	11	17.7
	61- 90 day	4	6.5
	>90 day	8	12.9
	Total	62	100.0

**Table 4:** Patient characteristics and associated factors of severity of burn pain at Yekatit 12 Hospitals, Addis Ababa, Ethiopia, from January 1, 2017, to March 30, 2017, G.C. (N = 62).

Variables		Severity of pain		Odds Ratio		P Value	95%C.I
		moderate	severe	Crude	Adjusted		
Age	1-14 years	1	40			0.039*∗*	0.009,0.883
	≥15 years	7	14	0.050	0.090		

Sex	Male	6	18			0.102	0.526, 20.452
	Female	2	36	6.000	3.278		

Degree of burn	Second	3	43			0.072	0.036, 1.155
	Third	5	11	0.153	0.204		

TSAB	<25%	6	9			0.033*∗*	1.184,51.043
	>25%	2	45	15.00	7.773		

Anxiety	Yes	5	45	0.248	0.262	0.151	0.042, 1.629
	No	3	7				

## Data Availability

The data used to support the findings of this study are available from the corresponding author upon request.

## Abbreviations

AAU: Addis Ababa University
NSAIDS: Nonsteroidal Anti-inflammatory Drugs
TBSA: Total burns surface area
PTSD: Posttraumatic Stress Disorder.
